# Changes in sleep patterns in primary care workers during the first wave of the COVID-19 pandemic in 2022 in Shanghai: a cross-sectional study

**DOI:** 10.1038/s41598-024-61311-z

**Published:** 2024-05-29

**Authors:** Wenshu Cao, Xiaoting Li, Yini Yan, Jianfeng Zhou, Jizhong Ye, Qiwei Lv

**Affiliations:** 1 Tianlin Community Health Center of Xuhui District, 500 Liuzhou Road, Shanghai, China; 2 Nanhui New Town Community Health Service Center of Pudong New Area, 280 Chaohe Road, Shanghai, China

**Keywords:** COVID-19, Sleep, Primary health care, Depression, Burnout, Insomnia, Health care, Medical research

## Abstract

The coronavirus disease (COVID-19) pandemic has significantly affected the sleep health of healthcare workers (HCWs); however, no studies have assessed this effect in primary HCWs. This cross-sectional, web-based study explored the prevalence and factors associated with sleep disorders among primary HCWs during the first COVID-19 outbreak in Shanghai from 12 July to 15 August 2022. Sociodemographic and work-related characteristics, various sleep dimensions, and exposure to patients with COVID-19 were assessed. They were screened for common mental disorders (depression, burnout, and stress). Overall, 313 primary HCWs were recruited. At least one sleep dimension in 84% of respondents deteriorated compared with that observed pre-pandemic; sleep quality (decline of 66%) and daytime sleepiness (increase of 56%) were the most affected domains. After excluding 145 primary HCWs with pre-pandemic ‘poor sleep’, depression (odds ratio [OR] 3.08; 95% confidence interval [CI] 1.59–5.98), weekly burnout symptoms (OR 2.57; 95% CI 1.32–5.03), and high psychological stress (OR 4.51; 95% CI 2.09–9.72) were associated with poor sleep patterns during the pandemic. After adjusting for significant differences between groups, for every 1-point increase in the Perceived Stress Scale score, an associated 12% increased risk of poor sleep (adjusted OR 1.12; 95% CI 1.05–1.21; p = 0.002) was observed. Most primary HCWs showed significant worsening of sleep quality, with increases in daytime sleepiness during the first wave of the COVID-19 pandemic in Shanghai. HCWs with high stress levels were at greater risks of sleep disorders.

## Introduction

Public health emergencies often deprive healthcare workers (HCWs) of sleep, in terms of time and quality, contributing to psychological problems such as generalised anxiety disorder and depression^1^. This problem may have been magnified among primary HCWs because they were responsible for the screening and diagnosis of patients with coronavirus disease (COVID-19) and providing frequent non-hospital/home care to the majority of patients with COVID-19. During the pandemic, HCWs also continued working despite the initial moments of collapse in the healthcare system^2^. Several primary care practitioners assumed new positions and responsibilities that eventually led to poor sleep health. A recent study reported that during the COVID-19 pandemic, < 6 h of sleep per day for general practitioners was a risk factor for an alteration in one’s mental health^3^. Sleep deprivation and burnout are not only linked to patient-related medical errors but also can lead to chronic depression, substance abuse, and suicide among medical professionals^4,5^. Notably, owing to the recurrent outbreaks of COVID-19, primary HCWs compared with other HCWs in China are being affected by sleep disorders for various reasons such as stress from the high risk of contracting the infection and transmitting it to their close contacts and changing professional obligations^3^. Some studies have found increased sleep disturbance and burnout rates among frontline HCWs only a few weeks after the response against COVID-19 was initiated^6,7^. In 2022, the COVID-19 Omicron variant caused the most severe and widespread outbreak in Shanghai since the pandemic began, causing a 3-month strict lockdown and massive COVID-19 testing and surveillance. That period was essentially Shanghai’s first wave of the pandemic. Furthermore, the impact of this pandemic in Shanghai on the sleep quality and psychological well-being of frontline primary HCWs, who are the mainstay of the fight against the epidemic, could be persistent.

To our knowledge, no study has explicitly focused on changes in the sleep patterns of primary HCWs during the epidemic period in China. Most studies have investigated the impact of pandemics on frontline staff in different roles in acute care and hospitals^8,9^. The duration of the COVID-19 pandemic is unknown, and primary HCWs in community hospitals are considered a significant force in the long-term response to the epidemic through their role as ‘gatekeepers’, which also means this group is the most vulnerable to sleep disorders. Identifying subgroups of primary HCWs susceptible to sleep disorders and identifying their characteristics are necessary to improve sleep quality and reduce the incidence of mental illness in this group. Good sleep may be associated with improved vaccination outcomes^10^. Similarly, reduced sleep, especially shorter than 7–9 h, may increase the risk of upper respiratory tract infections and related potential health outcomes such as metabolic syndrome, coronary heart disease, hypertension, diabetes, and impaired neurobehavioral performance^11^. In China, repeated outbreaks require primary HCWs to constantly switch roles, potentially affecting their sleep patterns and mental health. Thus, this observational cross-sectional study aimed to describe changes in multiple aspects of sleep among primary HCWs in Shanghai, China during the first wave of the COVID-19 epidemic in 2022.

## Results

### Survey population

We distributed 557 questionnaires, and 375 primary HCWs (response rate of 67.3%) responded; 59 primary HCWs were excluded because they did not meet the inclusion criteria (non-Shanghai area questionnaires) and three were excluded because they answered fewer than two questions. A total of 313 qualified questionnaires were eligible for analysis.

The median age of our sample (313 respondents) was 37 years (interquartile range [IQR] 32–42 years), and the majority of our respondents were females (77.6%). The proportion of Shanghai-origin respondents was 62.3%. Over three-quarters of respondents (77.6%) were married and 67.1% were the primary caretaker for at least one child. The sample mainly included physicians (40.9%) and nurses (39.3%). Regarding job level, the majority of the respondents (91.4%) had junior and mid-level titles. Table 1 lists the roles of the primary HCWs who participated in this study. Furthermore, 262 (83.7%) survey respondents reported having two or more frontline workplaces during the pandemic. The top five job locations were enclosed communities (71.2%), outpatient clinics (55.0%), quarantine hotels (46.0%), inpatient wards (36.7%), and Fangcang shelter hospitals (8.0%, they are large-scale temporary hospitals built in public venues).

**Table 1:** Demographics of Shanghai primary healthcare workers who responded to a survey on sleep health during the first wave of the COVID-19 pandemic in 2022.

	All participants (n = 313)
Age, median (IQR)	37 (32–42)
Sex, n (%)	304
Male	68 (22.4%)
Female	236 (77.6%)
Origin, n (%)	304
Origin Shanghai	195 (64.1%)
Origin Non-Shanghai	109 (35.9%)
Current level	
Junior	121 (38.7%)
Intermediate	165 (52.7%)
Senior	27 (8.6%)
Relationship condition, n (%)	304
Single	43 (14.1%)
Unmarried but dating	20 (6.6%)
Married	236 (77.6%)
Divorced	5 (1.6%)
Number of family children, n (%)	313
0	103 (32.9%)
1	173 (55.3%)
2	37 (11.8%)
Age category of children, n (%)	
Neonate/infant (< 1 year)	8 (3.9%)
Toddler/preschooler (1–4 years)	45 (21.8%)
School-age (5–12 years)	79 (38.3%)
Adolescents (12–18 years)	58 (28.2%)
Adults (> 18 years)	44 (21.4%)
Primary healthcare role, n (%)	313
Physician	128 (40.9%)
Nurse	123 (39.3%)
Hospital administrator	17 (5.4%)
Pharmacist	11 (3.5%)
Radiology Technologist	8 (2.6%)
Logistic staff	6 (1.9%)
Rehabilitation Technician	5 (1.6%)
Other: Maternal and child health worker (n = 3), Public Health Worker (n = 2), social worker (n = 1), unspecified (n = 9)	15 (4.8%)
Job locations, n (%)	
Enclosed communities	223 (71.2%)
Outpatient clinics	172 (55.0%)
Inpatient wards	209 (36.7%)
Quarantine hotels	144 (46.0%)
Fangcang shelter hospitals	25 (8.0%)
Administration office	7 (2.2%)
Centers for Disease Control and Prevention	6 (1.9%)

Data are presented as median (interquartile range) or n (%). *IQR* Interquartile range.

### Sleep patterns before and during the outbreak

**Figure 1:**
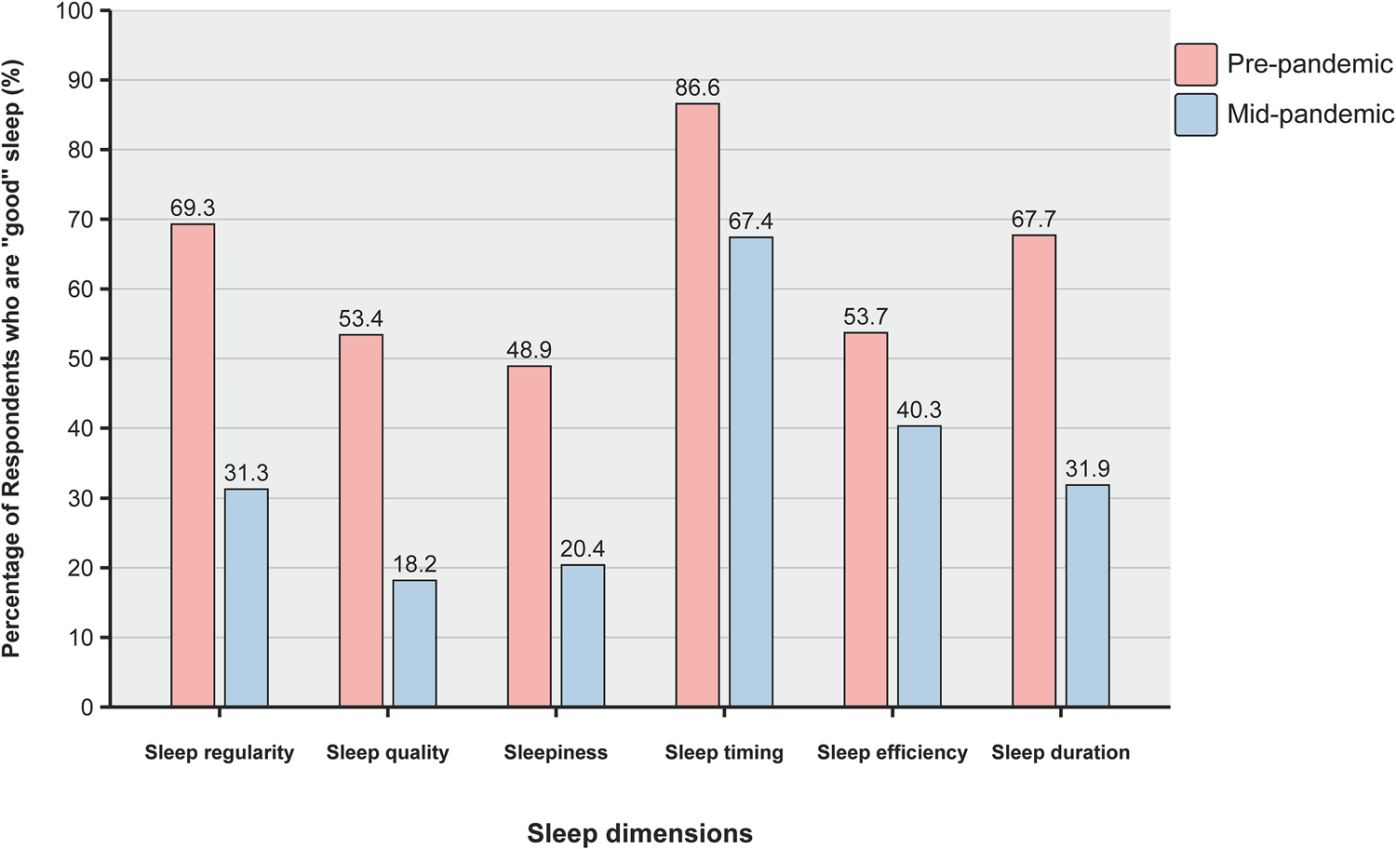
Percentage of respondents who reported good sleep habits (‘Often’, ‘Usually’, or ‘Always’) in each sleep dimension before and during the COVID-19 epidemic. Chi-square test showed that all the differences between before and during the epidemic were statistically significant, p < 0.001.

Before the pandemic, most primary HCWs experienced good (‘Often’, ‘Usually’, or ‘Always’) sleep regularity (69.3%), quality (53.4%), timing (86.6%), efficiency (53.7%), and duration (73.3%). Half (48.9%) of the respondents reported ‘often’ staying awake throughout the day. However, the proportion of respondents with regular healthy sleep patterns decreased significantly during the pandemic (p < 0.001; Fig. 1).

**Figure 2:**
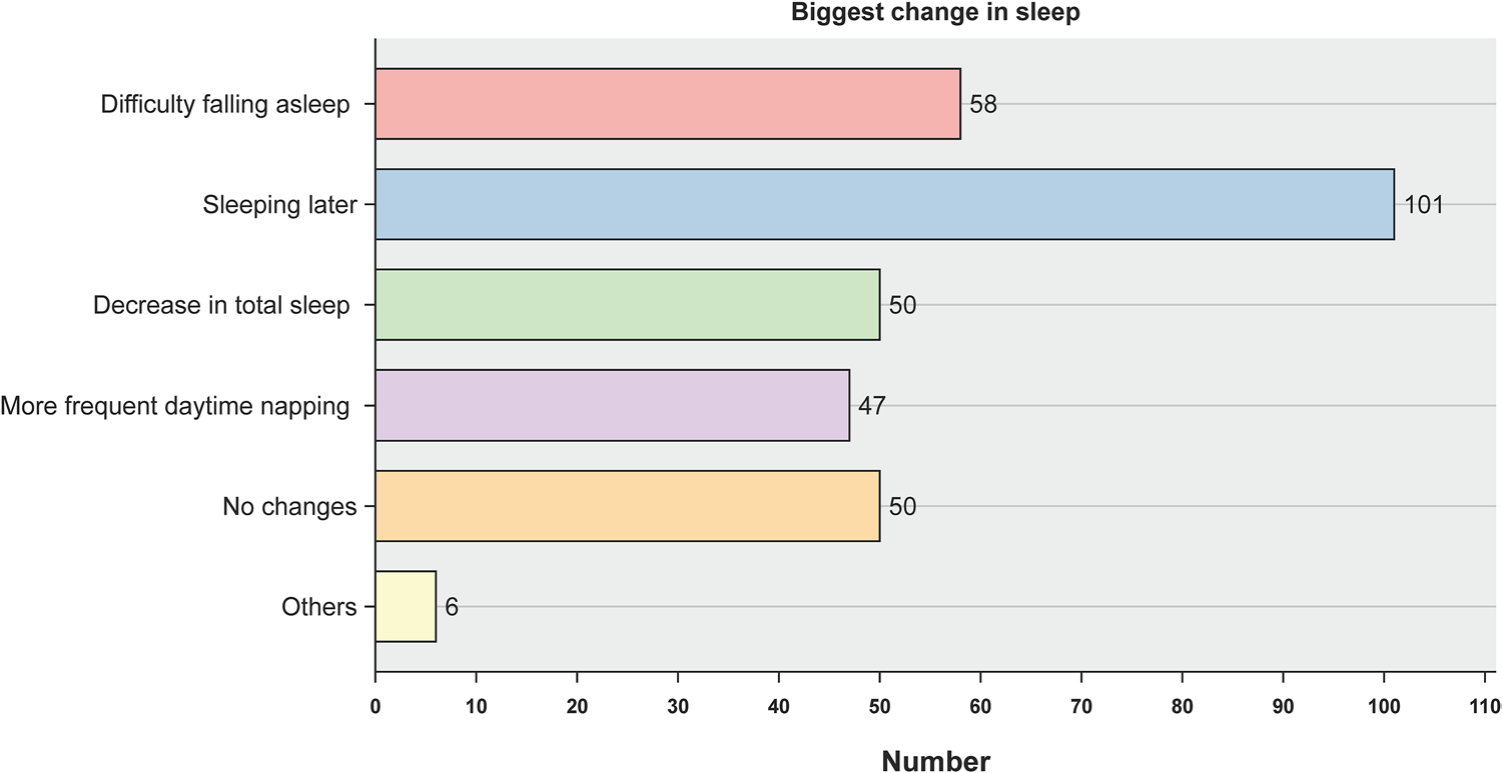
Proportion of respondents who reported the highest change in sleep patterns.

**Figure 3:**
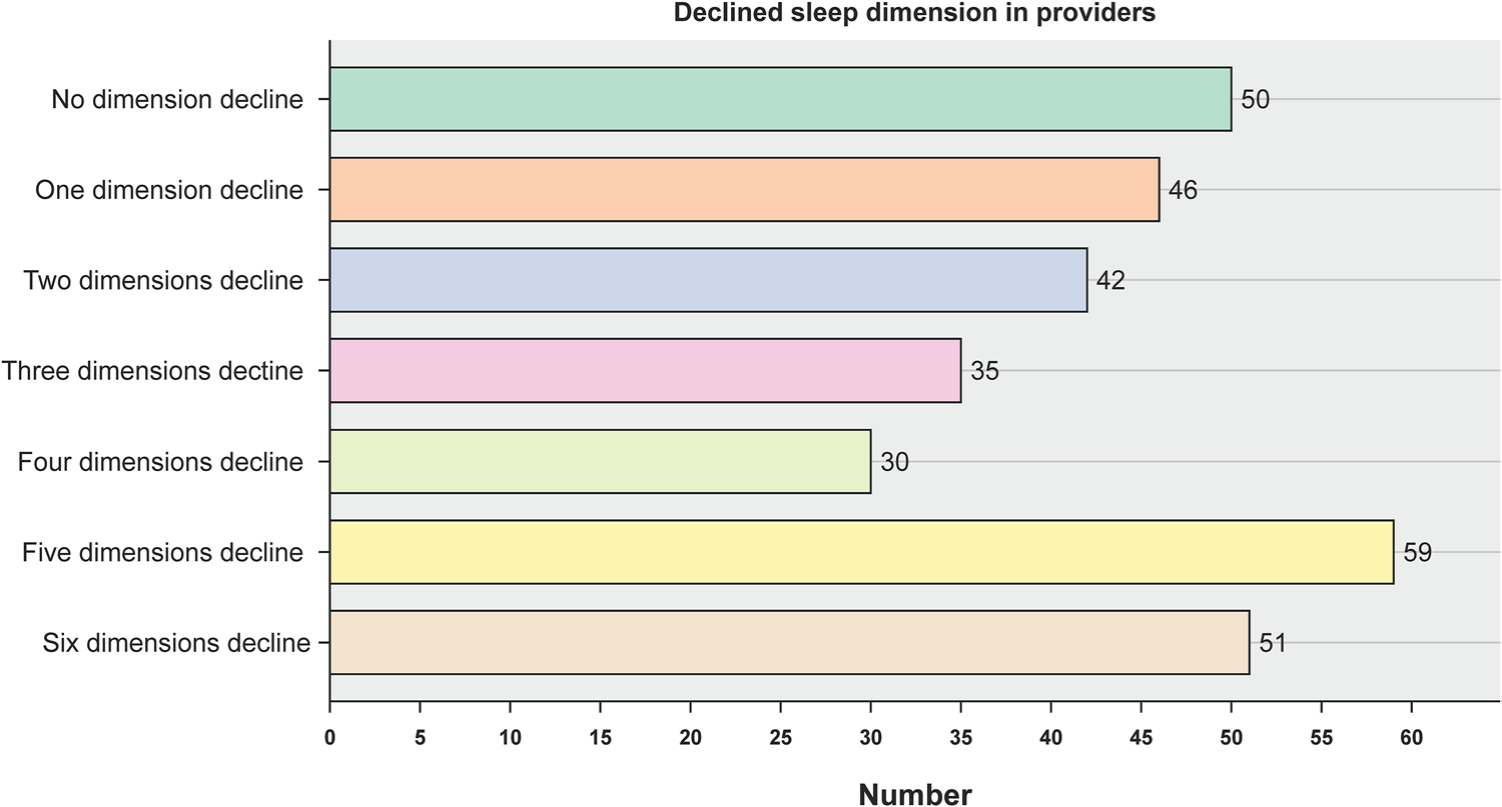
Proportions of respondents who showed a decline in sleep dimensions from the pre-pandemic levels.

Figure 2 shows the proportion of respondents who reported the largest change in sleep patterns; nearly a third (32.3%) complained of sleeping later than the pre-pandemic time. Over four-fifths of primary HCWs (84.0%) showed a decline in at least one sleep dimension from the pre-pandemic levels, and over two-thirds (69.3%) showed a reduction in at least two dimensions; 16.3% of the individuals showed a decline in all six domains (Fig. 3).

‘Sleep quality’ and ‘daytime sleepiness’ had the most significant deterioration from the pre-pandemic levels. As the pandemic continued, more than half of the primary HCWs in this study (55.3%) experienced varying degrees of decline in ‘sleep quality’. The proportion of HCWs who ‘often’ had good sleep quality decreased from 53.4 to 18.2%, showing a reduction of 65.9% (p < 0.001). Before the pandemic, 51.1% of the survey respondents had already experienced chronic excessive daytime sleepiness, and this proportion reached a staggering 79.6% during the pandemic, showing an increment of 55.7% (p < 0.001). Additionally, 48.2% of the participants reported an increase in daytime sleepiness frequency during the pandemic.

After excluding 145 (46.3%) HCWs with composite sleep health score (CSHS) < 4 before the pandemic, we divided the remaining 168 HCWs into the intra-pandemic good sleep group (n = 58, 34.5%) and poor sleep group (n = 110, 65.5%) for a comparative analysis. Table 2 shows the basic information of the primary HCWs with good and poor intra-pandemic sleep behaviours. No differences were observed in sex, medical roles, and ranking levels between the groups (p > 0.05). Participants with poor intra-pandemic sleep behaviours had significantly lower CSHS than those with good sleep habits (p < 0.001). Primary HCWs with high Perceived Stress Scale (PSS) scores (p < 0.001), especially scores > 28 (odds ratio [OR] 4.51; 95% confidence interval [CI] 2.09–9.72), had a higher likelihood of developing poor sleep patterns. Furthermore, those with weekly burnout symptoms had considerably increased odds of poor sleep during the pandemic (OR 2.57; 95% CI 1.32–5.03; Table 3) After adjusting for significant differences between groups, a multivariable analysis was conducted. We found that a higher PSS score was the only factor independently associated with increased risk of poor sleep during the pandemic (adjusted odds ratio [aOR] 1.12; 95% CI 1.05–1.21).

**Table 2:** Comparison of basic information on primary healthcare workers with good and poor sleep behaviours during the pandemic.

	Poor intra-pandemic sleep behaviours (n = 110)	Good intra-pandemic sleep behaviours (n = 58)	*p *value
Median age, median (IQR)	38 (32–42)	39 (32–44)	0.953
Sex, n (%)			0.734
Male	24 (22%)	14 (25%)	
Female	84 (78%)	43 (75%)	
Origin, n (%)	108	57	0.083
Origin Shanghai	67 (62%)	43 (75%)	
Origin Non-Shanghai	41 (38%)	14 (25%)	
Primary healthcare role, n (%)	110	58	0.924
Physician	40 (36%)	25 (43%)	
Nurse	41 (37%)	20 (34%)	
Hospital administrator	7 (6%)	3 (5%)	
Pharmacist	4 (4%)	3 (5%)	
Radiology technologist	4 (4%)	2 (3%)	
Others	14 (13%)	5 (9%)	
The current level, n (%)	110	58	0.378
Junior	39 (35%)	22 (38%)	
Intermediate	58 (53%)	33 (57%)	
Senior	13 (12%)	3 (5%)	
Relationship condition, n (%)	108	57	0.938
Single	12 (11%)	5 (9%)	
Unmarried but dating	7 (6%)	4 (7%)	
Married	86 (80%)	47 (82%)	
Divorced	3 (3%)	1 (2%)	
How the epidemic has altered one’s occupation, n (%)	110	58	
Increased work	99 (90%)	49 (84%)	0.294
Changed principal workplace	83 (75%)	38 (66%)	0.173
Changed job category	45 (41%)	23 (40%)	0.875
At least one child for which he or she is the primary carer, n (%)	77/110 (70%)	38/58 (66%)	0.552
Pandemic changed child care practice	63/110 (57%)	32/58 (55%)	0.794
Increased family burden	60/110 (55%)	27/58 (47%)	0.324
Daily COVID exposure	36/110 (33%)	17/58 (29%)	0.651
Respondents (or colleagues) COVID-positive, n (%)	80/110 (73%)	42/58 (72%)	0.965

Data are presented as median (interquartile range) or n (%). *IQR* interquartile range.

**Table 3:** Comparison of sleep and mental health between primary healthcare workers with good and poor sleep behaviours during the pandemic.

	Poor intra-pandemic sleep behaviours (n = 110)	Good intra-pandemic sleep behaviours (n = 58)	*p* value	Odds ratio of having poor pandemic sleep health
Pre-pandemic CSHS, mean ± SD	4.8 ± 0.6	4.9 ± 0.5	0.228	
Intra-pandemic CSHS, mean ± SD	2.9 ± 0.6	4.7 ± 0.5	< 0.001	
Pre-pandemic anxiety, n (%)	23 (21%)	9 (16%)	0.398	
Pre-pandemic depression, n (%)	12 (11%)	6 (10%)	0.911	
Pre-pandemic sleep apnoea, n (%)	13 (12%)	3 (5%)	0.163	
Pre-pandemic insomnia, n (%)	21 (19%)	5 (9%)	0.074	OR 2.50 (95% CI 0.89–7.03, p = 0.082)
Intra-pandemic depression screened positive, n (%)	31 (28%)	5 (9%)	< 0.001	OR 3.08 (95% CI 1.59–5.98, p = 0.001)
Burnout symptoms weekly, n (%)	59 (54%)	18 (31%)	0.005	OR 2.57 (95% CI 1.32–5.03, p = 0.006)
Average PSS score, mean ± SD	37.6 ± 9.8	27.5 ± 11.4	< 0.001	OR 1.10 (95% CI 1.06–1.15, p < 0.001)
PSS score > 28, n (%)	96 (87%)	35 (60%)	< 0.001	OR 4.51 (95% CI 2.09–9.72, p < 0.001)
Caffeinated beverage intake within 12 h of bedtime (often-always)	27 (25%)	7 (12%)	0.056	OR 2.37 (95% CI 0.96–5.84, p = 0.061)
Prolonged viewing of electronic device screens at night (often-always)	64 (58%)	26 (45%)	0.099	OR 1.71 (95% CI 0.90–3.25, p = 0.100)

Data are presented as n (%) or mean (standard deviation), unless otherwise specified. *CSHS* Composite sleep health score, *SD* Standard deviation, *PSS* Perceived stress scale.

The respondents who reported pre-pandemic insomnia (OR 2.50; 95% CI 0.89–7.03), caffeinated beverage intake ‘often’ within 12 h of bedtime (OR 2.37; 95% CI 0.96–5.84). and prolonged viewing of electronic device screens ‘often’ at night (OR 1.71; 95% CI 0.90–3.25) tended to sleep poorly, but this was not statistically significant. Furthermore, no significant statistical differences were observed in the pre-pandemic CSHS score, anxiety, depression, or sleep apnoea between those with good and poor pandemic sleep behaviours (Table 3).

### Impact of the pandemic on primary HCWs’ lives and careers

The respondents’ opinions of the impact of COVID-19 are summarised in Table 4. Self-reported pandemic adjustments in job requirements were almost universal (98.4% of respondents), and challenging situations were also frequently reported (97.8%); 89.4% of respondents were assigned SARS-CoV-2 nucleic acid testing and night duties. Over two-thirds (70.3%) of respondents were concerned about closure-induced difficulties in the case of unexpected sickness and lack of access to medical care for themselves or for family.

**Table 4:** Description of how COVID-19 affected the lives and careers of primary healthcare workers during the first wave in Shanghai in 2022.

	All respondents (n = 313)
What's changed in one's job in the first wave of the 2022 Shanghai COVID-19 pandemic? (multiple-choice questions)	n = 313
Increased working hours	271 (86.6%)
Decreased working hours	1 (0.3%)
Changed the primary workplace	239 (76.4%)
Changed the nature of primary work	133 (42.5%)
No change	5 (1.6%)
Others	6 (1.9%)
What difficulties have the first wave of the 2022 Shanghai COVID-19 pandemic caused you? (multiple-choice questions)	n = 313
More domestic responsibilities	174 (55.6%)
Frequent SARS-CoV-2 nucleic acid testing duties and occasional night attendance	281 (89.8%)
Concern about unexpected sickness and difficulties in accessing medical care for self/family	220 (70.3%)
Financial challenges	108 (34.5%)
Increased emotional stress	114 (36.4%)
No personally difficulty	7 (2.2%)
Others	9 (2.9%)
Respondents who care for at least one child at home	n = 210
Changed the child care management plan	180 (85.7%)
Additional adult to assist with child care in the family	129 (61.4%)
Spending more than 30 min daily assisting your children with educational responsibilities	80 (31.8%)
Change of residence because of concern about children or older adults being infected	116 (55.2%)
Intra-pandemic mental health issues	
Burnout symptoms weekly	146 (46.6%)
Depression screened positive	173 (55.3%)
The average score on the 14-items PSS	35.4 ± 11.1
PSS > 28 (moderate-to-severe stress)	255 (81.5%)
Frequently having nightmares	43 (13.7%)
Daytime habits reported as ‘often’ or ‘usually’, or ‘always’, which may impact sleep	
Caffeinated beverage intake within 12 h of bedtime	65 (20.8%)
Drinking alcohol within 6 h before bedtime	11 (3.5%)
Smoking within 6 h before bedtime	16 (5.1%)
Prolonged viewing of electronic device screens at night	179 (57.2%)

Data are reported as mean ± SD or n (%). *PSS* Perceived stress scale, *SD* Standard deviation.

Over a fifth (23.3%) of respondents showed positive results when screened for depression. The average PSS score was 35.4 ± 11.1, and 81.5% of respondents scored at least 29 points, which indicates moderate-to-severe stress. Additionally, about one-fifth (20.8%) of respondents ‘often’ took caffeinated beverages 12 h before bedtime, and more than half (57.2%) of the participants would frequently view electronic devices for prolonged periods at night.

Moreover, 85.7% of the respondents with children (n = 210) reported a loss of resources for childcare due to the epidemic. Over half (55.2%) of respondents changed their residence because of concerns about children or older adults being affected by COVID-19.

### Contact with patients or colleagues with confirmed COVID-19 status

Over half (58.8%) of the respondents reported weekly or daily exposure to patients with COVID-19; 229 (73.2%) respondents stated that their colleagues had been diagnosed with COVID-19. A significantly higher proportion (p < 0.001) of nurses (95/123,77.2%) than physicians (84/128, 65.6%) were diagnosed with COVID-19.

## Discussion

To our knowledge, this study is the first to investigate multidimensional sleep patterns in primary HCWs during the first wave of the 2022 COVID-19 pandemic in Shanghai. Other studies in China have generally focused on sleep as a secondary component of overall mental health^3^ or specific provider groups, such as HCWs from tertiary medical centres^12^, intensive care unit practitioners^13^, nurses^14^, and anaesthesiologists^15^. Benedict et al.^5^ investigated the sleep patterns of HCWs and non-HCWs using various sleep dimensions. However, sleep disturbances were more prevalent among frontline HCWs than among non-frontline and non-medical staff^16^. Primary HCWs play a critical role in the early identification and containment of the pandemic. Regarding overall sleep health, the frontline group should be the most considered^17^. According to our findings, the COVID-19 outbreak significantly influenced the sleep health (six dimensions) of primary HCWs. Sleep quality and daytime sleepiness were the most strongly affected aspects. Individuals with sleep disturbances had self-reported depression, weekly burnout symptoms, and high psychological stress (moderate-to-severe).

Our study included a larger group of primary HCWs with poor sleep habits than that in other studies^18^. The pre-pandemic and mid-pandemic rates of poor sleep health (46.3% and 78.6%, respectively) were considerably higher in our study than those reported in another study in the early stages of the COVID-19 outbreak in China^19^. The continuation of the pandemic may be having an adverse impact on the sleep health of primary HCWs. Similar to our findings, pandemic-related rates of depression^20^ and burnout^21^ among primary HCWs have been routinely reported to be more than 50%, while provider stress levels have been observed to exceed 80%.

As expected, high stress, burnout, and depression were not directly related to sex or medical roles, but they directly affected holistic sleep patterns. A possibility of the reciprocal influence of psychological factors on sleep disturbances may exist. On the one hand, the stress associated with a high risk of viral infection, perceived physical isolation, need for constant awareness of infection control protocols, and concerns about the well-being of family members may lead to depression and altered sleep patterns. On the other hand, poor sleep can result in daytime weariness, burnout, impairment of daily performance, and increased risk of crucial errors at work, all of which affect the psychological condition of HCWs^22^. Furthermore, a downward trend was observed in sleep-related disorders among primary HCWs in one to four sleep dimensions. In contrast, a sharp increase in such disorders was noted when more than five dimensions were involved. This phenomenon could be explained by a significant breakthrough effect when several sleep dimensions interact substantially.

Our findings contradict numerous previous conclusions that females, nurses, and frontline HCWs were the most consistently affected by COVID-19^3,5,18,23^. We did not observe significant differences between sexes, medical roles, or levels. A potential explanation is that almost all of the primary HCWs were involved in frontline roles with an equal workload during the peak time of the outbreak. Furthermore, many appeals were made to ensure that females and nurses receive increased attention and care during the pandemic, which may have increased the workload of male primary HCWs. Our research showed that mental health (stress, burnout, and depression) had a more robust association with sleep behaviour than that observed with job role or sex. Notably, providers with a high PSS score were disproportionately affected by sleep loss, indicating that focusing on psychological ailment interventions to improve sleep patterns may be particularly fruitful.

Ongoing public health crises can have immediate and long-term effects on the sleep and mental health of frontline primary HCWs; hence, it is important to establish proactive systems to assess and monitor the psychological well-being of various professionals in primary care and provide psychological support^18,24,25^. Although providers experiencing psychological stress may contract COVID-19^26^, the addition of poor sleep health to emotional stress has been associated with reduced immunity and poor vaccine response^27^. One study also revealed that insufficient sleep before and during the pandemic was related to an increased risk of COVID-19^28^. Regrettably, studies on earlier pandemics have suggested that the mental health impact on frontline primary HCWs was long lasting. However, the duration of any effect on provider sleep patterns is uncertain, and interventions should be provided to promote sleep health since it can be modified via self-management methods and organisational and work environment adjustments^18,29^. Longitudinal studies are required to examine the impact of the pandemic on sleep behaviour over time and to determine the elements that can predict the occurrence of sleep disorders.

This research had several limitations. First, participation was voluntary, which may have introduced an unpredictable bias owing to the self-selection of survey respondents. The risk of bias is particularly high when the response rate is low (67.3%), although this constraint is inherent to the approach employed and has been reported in other studies based on telematic surveys^30^. Second, a disadvantage of the convenience sample method was that it resulted in a limited sample size; hence, the sample lacked the statistical power to allow the evaluation of data from underrepresented healthcare positions, such as logistic staff and social workers. In reality, almost all (88%) of the studies included in large-sample systematic review and 89% of the studies in another large systematic review assessing mental health or sleep disorders related to pandemics were cross-sectional and employed convenience sampling^31,32^ Third, the variable selection process based on univariate p-values is commonly used in the literature to determine which variables to include in a logistic regression model; however, this may lead to overfitting and increased false positive rates. The implications of this approach may have consequences on the interpretation of the results and the generalizability of the findings.

In conclusion, during the first wave of the COVID-19 outbreak in 2022 in Shanghai, primary HCWs experienced a deterioration in various sleep parameters, with self-reported ‘sleep quality’ and ‘daytime sleepiness’ being the most strongly affected domains. Self-reported depression, weekly burnout symptoms, and high psychological stress were associated with poor sleep health during the COVID-19 pandemic. Furthermore, high stress was associated with higher odds of poor sleep health among the HCWs during the onset of the COVID-19 pandemic.

## Methods

### Study design

This anonymous, cross-sectional survey was performed via the Tencent questionnaire website (Tencent questionnaire: http://wj.qq.com). Written informed consent was obtained from each participant before the study began. This study was approved by the Ethics Committee of Shanghai Tianlin Community Health Center (Approval No: 2022-003) and in accordance with the Declaration of Helsinki. All the data were collected without any personal identifiers. In this study, medical personnel were asked to join a WeChat group where they were briefed on the purpose, nature, and administrative procedures of the study. The members of the group accepted the invitation to complete the survey anonymously.

Sample size was calculated using the following formula: N = u × P × (1 − P)/δ^2^, with u = 1.96 and its associated 95% confidence interval. According to a previous study, P represents a 15% prevalence rate with a 5% expected error rate. The least sample size was calculated as N = 1.96 × 0.15 × (1–0.15)/0.05^2^ = 100.

The survey included all primary HCWs (general practitioners, nurses, pharmacists, medical technicians, administrative staff, and logistics staff) in Shanghai community hospitals. Participants residing outside Shanghai area were excluded from the survey. Respondents who replied to fewer than two questions were disqualified. The survey questionnaire (ID: 10511743) was filled by scanning a pre-generated quick response (QR) code. The QR code was first sent by email to the person in charge of the target community hospital, who then sent it to the respondents via a mass email. The survey was opened between 12 July and 15 August 2022. No personalised invitations were made as this survey was designed to capture a target sample size, and participation was entirely voluntary.

The COVID-19 epidemic in Shanghai had been effectively controlled in the period during which the survey link was open. However, as stringent management measures continued, the workload of primary HCWs on the frontline remains heavy.

### Survey content

The questionnaire contained questions on basic demographic information—survey respondents’ role, age, household location (Shanghai or non-Shanghai), sex, fertility, relationship status, and sleep patterns. The respondents also reported information on how the pandemic had changed the content and location of their jobs, children’s education in the household, and family responsibilities (see Supplementary Information 1 for details).

Referring to the study by Benedict et al.^5^, we used the CSHS to measure good or poor sleep patterns before and during the pandemic. The CSHS is a widely used multi‐dimensional indicator of sleep health, with higher values indicating good sleep health^5,33–35^. The CSHS, a newer assessment tool compared with the previously predominant Pittsburgh Sleep Quality Index, which mainly relies on individual subjective evaluations, may attempt to offer a more comprehensive perspective on an individual’s sleep health by incorporating both subjective reports and objective measurements. A 6-point Likert scale (1 = never; 2 = rarely; 3 = sometimes; 4 = often; 5 = usually; 6 = always) was used to quantify the frequency of sleep patterns before and during the pandemic, with higher scores indicating a higher frequency of healthy sleep patterns. The CSHS is the total sleep health score (maximum of 36 points) divided by the dimensions of the questions answered (maximum of 6). In the main survey content, the respondents were asked to report the frequency at which they exhibited sleep patterns in the following six dimensions^33^:

- Sleep regularity: ‘How often do you have a regular sleep time rhythm? (go to bed at the same time of night and wake up at the same time of the morning)’
- Sleep quality: ‘How often do you wake up refreshed?’
- Sleepiness: ‘How often do you stay awake throughout the day without nodding off or dozing off?’
- Sleep timing: ‘How often do you go to bed after midnight?’
- Sleep efficiency: ‘How often do you fall asleep within 30 min from the time you prepare to sleep (including falling back to sleep after short periods of being awake in the night)?’
- Sleep duration: ‘How often do you get 6–8 h of sleep per day?’

A score of 4 (i.e. ‘often’ for self-reported healthy sleep in all dimensions) was used as a cut-off point for ‘good’ and ‘poor’ sleep. To identify HCWs whose sleep were most affected by the pandemic, we excluded those who were ‘poor sleepers’ (with a CSHS of less than 4) before the pandemic and conducted a secondary analysis. The CSHS is more concise than other sleep scores, and its validity has been demonstrated in other population-based studies^34–37^.

In addition, an abbreviated version of the Maslach Burnout Scale^38^ was used to assess burnout. We purchased the Maslach Burnout Inventory license before administering the instrument via the MindGarden (http://www.mindgarden.com) webpage. The 14-item PSS^39^ and 2-item patient health questionnaire^40^ were used to evaluate stress and depression, respectively. A PSS score of more than 28 indicates moderate-to-severe stress^39^. Other factors affecting sleep frequency (e.g., time spent in front of a mobile phone screen, drinking alcohol, or consuming caffeine or smoking before bedtime) were also investigated. Finally, the respondents were asked about occupational exposure to COVID-19 and infection. All questions were not compulsory.

### Statistical analysis

Appropriate central tendency and dispersion measures or counts and percentages for categorical data were used to summarise the data. As this was an anonymous survey, replies were reviewed for plausibility, but data reliability could not be guaranteed. Mann–Whitney U or Kruskal–Wallis tests were used to compare continuous non-parametric data among categorical variables. Paired t-tests were used to compare parametric data from several time points for each participant. Categorical variables within groups were compared using chi-squared tests, whereas paired proportions were compared using McNemar chi-squared test. Parameters with p-values < 0.1 in the univariate analyses were included in the logistic regression. p-values < 0.05 were considered statistically significant. All statistical analyses were conducted using R 3.6.3 and Python 3.7.

### Supplementary Information

Supplementary Information 1.

Supplementary Information 2.

Supplementary Information 3.

Supplementary Information 4.

## Data Availability

Due to the nature of this study, participants in this study did not agree to share their data publicly and therefore supporting data could not be provided. If anyone would like to obtain data from this study, please contact the corresponding author.
