# Supplementary material for: Changes in sleep patterns in primary care workers during the first wave of the COVID-19 pandemic in 2022 in Shanghai: a cross-sectional study
Source: Sci Rep. 2024 May 29;14:12373. doi: 10.1038/s41598-024-61311-z (PMC11137069; doi:10.1038/s41598-024-61311-z)
Supplement: Supplementary file 1 — Supplementary Information 1. [file 41598_2024_61311_MOESM1_ESM.pdf]

For use by Wenshu Cao only. Received from Mind Garden, Inc. on April 24, 2024

Translations are provided as a courtesy with license purchase, and are subject to the terms of that license.

Chinese Translation only:

Maslach Burnout Inventory™

MBI-HSS, MBI-ES, MBI-GS, and MBI-GS (S) forms only

By Christina Maslach, Susan E. Jackson, Michael P. Leiter, Wilmar B. Schaufeli & Richard L. Schwab

Published by Mind Garden, Inc.
www.mindgarden.com

Important Note

It is your legal responsibility to compensate the copyright holder of this work — via payment to Mind Garden — for reproduction or administration in any physical or digital medium, including online survey, handheld survey devices, etc.

You agree to track the number of reproductions or administrations, and to compensate Mind Garden for any usage in excess of the quantity purchased.

This translations document is subsidiary to the License to Administer. All terms of the License to Administer apply also to this translations document.

This instrument, and any use thereof, is covered by U.S. and international copyright laws. For any further use or reproduction of the instrument, in whole or in part, contact Mind Garden, Inc.

MBI-Human Services Survey: Copyright ©1981 Christina Maslach & Susan E. Jackson.
MBI-Human Services Survey for Medical Personnel: Copyright ©1981, 2016 Christina Maslach & Susan E. Jackson.
MBI-Educators Survey: Copyright ©1986 Christina Maslach, Susan E. Jackson & Richard L. Schwab.
MBI-General Survey: Copyright ©1996 Wilmar B. Schaufeli, Michael P. Leiter, Christina Maslach & Susan E. Jackson.
MBI-General Survey for Students: Copyright ©1996, 2016 Wilmar B. Schaufeli, Michael P. Leiter, Christina Maslach & Susan E. Jackson.
All rights reserved in all media. Published by Mind Garden, Inc., www.mindgarden.com

For use by Wenshu Cao only. Received from Mind Garden, Inc. on April 24, 2024

Translation Quality not Guaranteed

Mind Garden instrument translations are of varying quality. Many are done by researchers. Some translations are translated and then back-translated to check the quality, while others have not been back-translated. We typically do not know the dialect of the translation. We also do not have validation data on the translation. Some translations do not include all of the items that are on the English form; sometimes a scale or scales are missing on the translation. You will receive what we have with no warranty or assurance of quality or dialect. Basically, we try to provide you with what we have available to facilitate your work.

5. 如在工作中出现以下感觉，请选出您出现各种感觉的频率：

For use by Wenshu Cao only. Received from Mind Garden, Inc. on April 24, 2024

	从无	一年中很少几次或更少	每月一次或更少	每月几次	每周一次	每周几次	每天
(1)对于我的工作，我感觉心理疲惫	0	1	2	3	4	5	6
(2)一天的工作结束时，我感觉精疲力竭	0	1	2	3	4	5	6
(3)早晨起床时，我感觉疲惫，且不得不面对又一天的工作	0	1	2	3	4	5	6
(4)我容易理解病人对事情的感受	0	1	2	3	4	5	6
(5)我觉得自己对待一些病人就好像他们是物件而不是人	0	1	2	3	4	5	6
(6)整天上班要和人打交道对我来说真是很沉重的负担	0	1	2	3	4	5	6
(7)我处理病人的问题非常有效	0	1	2	3	4	5	6
(8)我感觉工作令我耗竭	0	1	2	3	4	5	6
(9)我觉得我积极地影响着别人的生活	0	1	2	3	4	5	6
(10)自从我从事这份工作以来，我对人变得缺少人情味	0	1	2	3	4	5	6
(11)我担心工作让我变得缺少人情味	0	1	2	3	4	5	6
(12)我感觉精力很充沛	0	1	2	3	4	5	6
(13)对于我的工作，我有挫折感	0	1	2	3	4	5	6
(14)我感觉我工作太努力	0	1	2	3	4	5	6
(15)我并不真正关心某些病人的情况	0	1	2	3	4	5	6
(16)工作中要直接和人打交道对我来说压力太大	0	1	2	3	4	5	6
(17)我很容易与病人建立轻松的氛围	0	1	2	3	4	5	6
(18)在工作中，我完成了很多值得做的事情	0	1	2	3	4	5	6
(19)在为病人做事时与病人配合良好，我会感觉很高兴	0	1	2	3	4	5	6
(20)我感觉智穷力竭，力不从心	0	1	2	3	4	5	6
(21)在工作中，我能平静地处理自己的情绪问题	0	1	2	3	4	5	6
(22)我觉得病人因为他们自身的一些问题而责备我	0	1	2	3	4	5	6

（修改自Maslach 疲溃调查表（Maslach Burnout Inventory），并取得版权所有者的同意。未经许可，不得转载使用）

MBI-General Survey: Copyright ©1996 Wilmar B. Schaufeli, Michael P. Leiter, Christina Maslach & Susan E. Jackson.
MBI-Human Services Survey: Copyright ©1981 Christina Maslach & Susan E. Jackson.
MBI-Educators Survey: Copyright ©1986 Christina Maslach, Susan E. Jackson & Richard L. Schwab.
All rights reserved in all media. Published by Mind Garden, Inc., www.mindgarden.com

For use by Wenshu Cao only. Received from Mind Garden, Inc. on April 24, 2024

教師調查*

以下是關於教師對教學工作及對其他教學人員所持的態度，請仔細閱讀這些關於工作上感受的敘述，決定你是否有同感。
如果你從沒有這種感受，請在答案紙上填上"0"。
如果你曾有這種感受，請填上"1"至"6"的數字以表示你「多少時候」有這種感受。

「 多少時候」：

從沒有	0
一年數次或少些	1
一月一次或少些	2
一月數次	3
一週一次	4
一週數次	5
每天都有	6

1. 工作使我覺得情感不斷消耗。
2. 每天工作後,我感到筋疲力盡。
3. 每早起來及知道要面對另一天的工作時,我已感到疲乏。
4. 我可以容易明白學生對事物的感受。
5. 我感到我對待一些學生好像他們是非人的物體。
6. 要整天與人在工作中交往，對我實在是一項負擔。
7. 我很有效地處理學生的問題。
8. 我因工作感到身心受損。
9. 我感到透過工作，我能積極地影響別人的一生。
10. 自從任職這工作，我對人已變得冷漠。
11. 我擔心這份工作已使我感情麻木。
12. 我感到精力非常充沛。
13. 我的職業使我有挫敗感。
14. 我感到我過份努力工作。
15. 我真的不在乎發生在一些學生身上的事情。
16. 要與人一起工作，直接給予我太大的壓力。
17. 和學生相處時，我可以很容易營造輕鬆的氣氛。
18. 在與學生緊密合作後，我常感到很興奮。
19. 在這工作上，我有很多有價值的成就。
20. 我感到好像身臨絕境。
21. 在工作上，我冷靜地處理情緒問題。
22. 我感到學生將他們的一些問題歸咎於我。

MBI-General Survey: Copyright ©1996 Wilmar B. Schaufeli, Michael P. Leiter, Christina Maslach & Susan E. Jackson.
MBI-Human Services Survey: Copyright ©1981 Christina Maslach & Susan E. Jackson.
MBI-Educators Survey: Copyright ©1986 Christina Maslach, Susan E. Jackson & Richard L. Schwab.
All rights reserved in all media. Published by Mind Garden, Inc., www.mindgarden.com

For use by Wenshu Cao only. Received from Mind Garden, Inc. on April 24, 2024

教 育 工 作 問 卷

本問卷的目的，在瞭解教育工作人員對自己的工作及對工作上有關的人之看法。本問卷有二十二題，都與您的工作感受有關。請仔細閱讀每一題目後，再決定您在工作上是否有此一感受。如果您從未曾有這樣的感受，請在該題前的空格上填答"0"（零）；如果您曾有此一感受，請依次數多寡選擇一個最能描述您感受程度的答案（由1至6，自選一數）。請參看下面例題：

例題：

感受程度：	0	1	2	3	4	5	6
	從未有	每年有少數幾次	每月有一次或少於一次	每月有幾次	每週一次	每週有多次	每天都有

感受程度 (0-6)　　題　　目：

________ 我感到工作情緒低落

如果您從未感到工作情緒低落，請在題目前空格上填答"0"（零）；如果您很少感到工作情緒低落（每年有少數幾次），請填答數字"1"…；如果您一直有工作情緒低落的感覺（每天都有），請填答"6"。

MBI-General Survey: Copyright ©1996 Wilmar B. Schaufeli, Michael P. Leiter, Christina Maslach & Susan E. Jackson.
MBI-Human Services Survey: Copyright ©1981 Christina Maslach & Susan E. Jackson.
MBI-Educators Survey: Copyright ©1986 Christina Maslach, Susan E. Jackson & Richard L. Schwab.
All rights reserved in all media. Published by Mind Garden, Inc., www.mindgarden.com

For use by [illegible] 2024

感受程度：	0	1	2	3	4	5	6
	從未有	每年有少數幾次	每月有一次或少於一次	每月有幾次	每週一次	每週有多次	每天都有

感受程度 (0-6)　　題　　目：

1. ________ 我覺得我的工作熱情已消耗殆盡。
2. ________ 下班時，我感到精疲力竭。
3. ________ 早晨醒來，想到又必須面對一天的工作，我就覺得厭倦。
4. ________ 我很容易體會學生對事物的感受。
5. ________ 我會忽略某些學生也是具人格的個體。
6. ________ 工作上整天與人相處，實在令我感到壓力沈重。
7. ________ 我能有效地處理學生的問題。
8. ________ 我已對我的工作感到很倦怠。
9. ________ 我覺得我的工作能積極地影響他人。
10. ________ 從事這項教育工作後，使我變得對人較冷漠。
11. ________ 我擔心我的工作使我愈來愈嚴酷。
12. ________ 我覺得精力很充沛。
13. ________ 我的工作使我有挫折感。
14. ________ 我覺得我工作得過份賣力了。
15. ________ 對某些學生，我不太在乎他們會發生什麼事情。
16. ________ 工作上直接與人接觸，給我很大的壓力。
17. ________ 與學生相處時，我很容易培養輕鬆的氣氛。
18. ________ 與學生一起工作後，我覺得很愉快。
19. ________ 從事這項教育工作，我做了許多有意義的事情。
20. ________ 我似乎對我的工作已費盡心思，到了一籌莫展的地步。
21. ________ 我能很冷靜地處理工作上的情緒問題。
22. ________ 我感到學生將某些問題歸咎於我。

謝謝您的合作！

Translation and modification are made by Tien-Suze Chou, 1989.

MBI-General Survey: Copyright ©1996 Wilmar B. Schaufeli, Michael P. Leiter, Christina Maslach & Susan E. Jackson.
MBI-Human Services Survey: Copyright ©1981 Christina Maslach & Susan E. Jackson.
MBI-Educators Survey: Copyright ©1986 Christina Maslach, Susan E. Jackson & Richard L. Schwab.
All rights reserved in all media. Published by Mind Garden, Inc., www.mindgarden.com

For use by Wenshu Cao only. Received from Mind Garden, Inc. on April 24, 2024

教師工作環境問卷

編印者：黃鴻文

親愛的國中教師：

在您的工作環境中，或許您會遭遇到某些工作壓力。這些壓力會影響您的健康和工作效率。本人曾任國中教師多年，深深體會工作壓力對教師的不良影響。因此我以教師的工作壓力為題，從事博士論文的研究，希望藉此研究結果，作為改善教師工作環境的依據，進而提升教師在學校的生活品質。

這份問卷目的在瞭解您所承受的工作壓力，和您對工作壓力的反應。填答問卷不需具名。所有資料僅供學術研究之用，對您個人不會有任何影響。如果您想知道研究結果，請將姓名、住址填在所附的空白紙上。本人非常樂意將研究結果通知您。

您的寶貴意見不僅使本研究獲得正確的結論，也能使本研究提出具體建議，茲以增進全體教師的福利。感謝您的合作。敬祝

健康快樂

美國德州大學奧斯汀校區
課程與教學系研究生

黃鴻文敬上
七十七年一月

MBI-General Survey: Copyright ©1996 Wilmar B. Schaufeli, Michael P. Leiter, Christina Maslach & Susan E. Jackson.
MBI-Human Services Survey: Copyright ©1981 Christina Maslach & Susan E. Jackson.
MBI-Educators Survey: Copyright ©1986 Christina Maslach, Susan E. Jackson & Richard L. Schwab.
All rights reserved in all media. Published by Mind Garden, Inc., www.mindgarden.com

第一部份

說明：這個部份的題目都是您在學校生活中，可能遭遇到的事情。請按這些事情「困擾」您的程度，在題目右方1，2，3，4，5中，「圈選」適當的號碼。

1＝從未困擾我
2＝很少困擾我
3＝有時困擾我
4＝常常困擾我
5＝總是困擾我

「例」超時間上班。　1　2　3　④　5

	從未困擾我	很少困擾我	有時困擾我	常常困擾我	總是困擾我
1.教學職權太少。	1	2	3	4	5
2.很難獲得改進教學的資料。	1	2	3	4	5
3.工作無法在學校作完，需要帶回家處理。	1	2	3	4	5
4.批改許多學生作業。	1	2	3	4	5
5.在校內不得不違背我的理想來教導學生。	1	2	3	4	5
6.不知道學校行政人員如何評量我的教學。	1	2	3	4	5
7.校內有關的決策我沒有表示意見的機會。	1	2	3	4	5
8.我的工作要配合升學的要求。	1	2	3	4	5
9.家長對子女的行爲疏於管教。	1	2	3	4	5
10.不能滿足家長的要求。	1	2	3	4	5
11.處理與其他老師的歧見。	1	2	3	4	5

For use by: Wenshu Cao only. Received from Mind Garden, Inc. on April 24, 2024

	從未困擾我	很少困擾我	有時困擾我	常常困擾我	總是困擾我
12.老師之間的閒言閒語。	1	2	3	4	5
13.無法滿足學生的期待。	1	2	3	4	5
14.接待家長來訪。	1	2	3	4	5
15.校園暴力事件。	1	2	3	4	5
16.品行不良的學生惹禍生事。	1	2	3	4	5
17.學生的吵鬧行爲。	1	2	3	4	5
18.處理學生之間的糾紛。	1	2	3	4	5
19.花費個人時間參加教學以外的學校活動。	1	2	3	4	5
20.學校行政人員的要求不一致。	1	2	3	4	5
21.參加各種會議花費太多時間。	1	2	3	4	5
22.處理和家長的歧見。	1	2	3	4	5
23.處理和學校行政人員的歧見。	1	2	3	4	5
24.學校行政人員處事不公。	1	2	3	4	5
25.沒有機會進修。	1	2	3	4	5
26.班級間的競爭被用來評量教師的能力。	1	2	3	4	5
27.學生程度參差不齊。	1	2	3	4	5
28.家長不關心學生學業。	1	2	3	4	5
29.工作量太多影響我的教學品質。	1	2	3	4	5
30.老師間的競爭增加我的工作量。	1	2	3	4	5
31.與我任教同一班級的某些老師能力太差。	1	2	3	4	5
32.作家庭訪問。	1	2	3	4	5

（請翻頁繼續作答）

MBI-General Survey: Copyright ©1996 Wilmar B. Schaufeli, Michael P. Leiter, Christina Maslach & Susan E. Jackson.
MBI-Human Services Survey: Copyright ©1981 Christina Maslach & Susan E. Jackson.
MBI-Educators Survey: Copyright ©1986 Christina Maslach, Susan E. Jackson & Richard L. Schwab.
All rights reserved in all media. Published by Mind Garden, Inc., www.mindgarden.com

第二部份

說明：這個部份的題目是關於您的學生、同事、家人……等對您的看法。請按照您「同意」的程度，在題目右方1，2，3，4，5中，「圈選」適當的號碼。

1 ＝不同意
2 ＝有點同意
3 ＝中等同意
4 ＝相當同意
5 ＝非常同意

	不同意	有點同意	中等同意	相當同意	非常同意
1.我的學生支持我的作法。	1	2	3	4	5
2.我的學生體諒我工作上的辛勞。	1	2	3	4	5
3.我的學生感激我的教導。	1	2	3	4	5
4.我的學生認爲我是一位好老師。	1	2	3	4	5
5.我的學生尊敬我。	1	2	3	4	5
6.學生家長支持我的作法。	1	2	3	4	5
7.學生家長體諒我工作上的辛勞。	1	2	3	4	5
8.學生家長感激我教導他們的子女。	1	2	3	4	5
9.學生家長尊重我。	1	2	3	4	5
10.學生家長體諒我工作上的難處。	1	2	3	4	5
11.我的家人願意傾聽我抱怨工作上的問題。	1	2	3	4	5
12.我的家人願意協助我完成某些工作（如批改考卷…等）	1	2	3	4	5
13.我的家人會讚賞我的工作成果。	1	2	3	4	5
14.我的家人關心我在學校工作的情形。	1	2	3	4	5

（請翻頁繼續作答）

For use by Wenshu Cao only. Received from Mind Garden, Inc. on April 24, 2024

	不同意	有點同意	中等同意	相當同意	非常同意
15.當我遭遇工作挫折，我的家人會安慰我。	1	2	3	4	5
16.校內其他老師願意傾聽我抱怨工作上的問題。	1	2	3	4	5
17.校內其他老師願意提供給我工作上的建議。	1	2	3	4	5
18.校內其他老師願意協助我解決工作上的問題。	1	2	3	4	5
19.校內其他老師會讚賞我的工作成果。	1	2	3	4	5
20.當我遭遇工作挫折，校內其他老師會安慰我。	1	2	3	4	5
21.學校行政人員願意傾聽我抱怨工作上的問題。	1	2	3	4	5
22.學校行政人員願意提供給我工作上的建議。	1	2	3	4	5
23.學校行政人員願意協助我解決工作上的問題。	1	2	3	4	5
24.學校行政人員會讚賞我的工作成果。	1	2	3	4	5
25.當我遭遇工作挫折，學校行政人員會安慰我。	1	2	3	4	5
26.學校發的薪水和我的工作份量相當。	1	2	3	4	5
27.學校發的薪水能提供適當的生活水準。	1	2	3	4	5
28.學校的辦公室的設備良好。	1	2	3	4	5
29.校內教師的休閒設備良好。	1	2	3	4	5
30.校內教師的衛生設備良好。	1	2	3	4	5
31.教師的福利令我滿意。	1	2	3	4	5

第三部份 → MBI

說明：這個部份的題目是想瞭解您在學校工作的感受。請按您心中的感受，在題目右方1，2，3，4，5中，「圈選」適當的號碼。

（請翻頁繼續作答）

MBI-General Survey: Copyright ©1996 Wilmar B. Schaufeli, Michael P. Leiter, Christina Maslach & Susan E. Jackson.
MBI-Human Services Survey: Copyright ©1981 Christina Maslach & Susan E. Jackson.
MBI-Educators Survey: Copyright ©1986 Christina Maslach, Susan E. Jackson & Richard L. Schwab.
All rights reserved in all media. Published by Mind Garden, Inc., www.mindgarden.com

1 ＝從未有這種感覺
2 ＝很少有這種感覺
3 ＝有時有這種感覺
4 ＝常常有這種感覺
5 ＝總是有這種感覺

	從未有這種感覺	很少有這種感覺	有時有這種感覺	常常有這種感覺	總是有這種感覺
1.教學工作使我情緒非常消沈。	1	2	3	4	5
2.上了一天課，我有一種消耗殆盡的感覺。	1	2	3	4	5
3.清晨醒來，想到必須面對另一天的工作，我就覺得好累。	1	2	3	4	5
4.我可以輕易瞭解學生心中的感覺。	1	2	3	4	5
5.我會把一些學生不當「人」看待。	1	2	3	4	5
6.成天和學生周旋，實在令我情緒緊張，難以鬆弛。	1	2	3	4	5
7.我能很有效地處理學生的問題。	1	2	3	4	5
8.教學工作使我心力交瘁。	1	2	3	4	5
9.透過教學工作，我能對學生的生活產生好的影響。	1	2	3	4	5
10.當了老師以後，我對學生漸漸變得冷漠無情。	1	2	3	4	5
11.我担心教學工作令我性情嚴厲。	1	2	3	4	5
12.我覺得精力充沛。	1	2	3	4	5
13.教學工作使我有挫折感。	1	2	3	4	5
14.我對教學工作太賣命了。	1	2	3	4	5
15.對某些學生，我不太在乎他們會變得怎麼樣。	1	2	3	4	5

（請翻頁繼續作答）

For use by Wenshu Cao only. Received from Mind Garden, Inc. on April 24, 2024

	從未有這種感覺	很少有這種感覺	有時有這種感覺	常常有這種感覺	總是有這種感覺
16.直接跟學生打交道，給我很大的壓力。	1	2	3	4	5
17.我很會醞釀輕鬆的氣氛跟學生相處。	1	2	3	4	5
18.跟學生一起解決問題後，我覺得很愉快。	1	2	3	4	5
19.在教學工作中，我已經完成許多很有價值的事情。	1	2	3	4	5
20.在教學工作上，我已到無計可施，一籌莫展的地步了。	1	2	3	4	5
21.我能很冷靜地處理工作上的情緒問題。	1	2	3	4	5
22.我想學生會將他們的某些難題，歸咎於我	1	2	3	4	5

（翻譯，修訂自 Human Services Survey*）

基本資料

(　　) 1.性別：(1)男(2)女

(　　) 2.任教年資：(1)1－2年(2)3－5年(3)6－10年(4)11年以上

(　　) 3.職務：(1)導師(2)專任老師

(　　) 4.任教科別：(1)語文科(2)社會科(3)數理科(4)藝能科(5)其他

(　　) 5.教育背景：(1)師專、師範學校畢業(2)師大師院畢業(3)一般專科畢業(4)一般大學畢業(5)其他

(　　) 6.婚姻：(1)未婚(2)已婚有小孩(3)已婚無小孩(4)其他

（謝謝您的合作）

* C. Maslach & S. Jackson: Human Services Survey. Copyright 1981, 1986 by Consulting Psychologists Press, Inc. 577 College Avenue, Palo Alto, CA 94036. Translated and modified by Horngwen Huang in December, 1987.

如果您想知道研究結果，請在所附的空白紙上填寫姓名地址！

MBI-General Survey: Copyright ©1996 Wilmar B. Schaufeli, Michael P. Leiter, Christina Maslach & Susan E. Jackson.
MBI-Human Services Survey: Copyright ©1981 Christina Maslach & Susan E. Jackson.
MBI-Educators Survey: Copyright ©1986 Christina Maslach, Susan E. Jackson & Richard L. Schwab.
All rights reserved in all media. Published by Mind Garden, Inc., www.mindgarden.com

For use by Wenshu Cao only. Received from Mind Garden, Inc. on April 24, 2024

教 學 工 作 環 境 問 卷

敬愛的教師們：

教育是一件重要的工作，無論在那一個國家，教師都是備受尊重的。不過目前教學環境所加給教師們的責任和壓力，都十分沈重。本項問卷調查係與美國萊特州立大學合作進行，研究者一方面是要瞭解我國教師對教學工作的感受和一般因應情況，並希望能根據所得資料向有關方面提供改善的建議，同時也將以這些資料和美國教師們的反應進行比較。

本問卷不需要署名，所有個人資料，除將用作統計分析外，絕不公開。

謝謝您的合作！

國立台灣師範大學教育心理與輔導系
黃堅厚 謹啓

"Research Edition Translation performed by **Chi-en Hwang** on this date **December 1, 1997.**
MBI-General Survey: Copyright ©1996 Wilmar B. Schaufeli, Michael P. Leiter, Christina Maslach & Susan E. Jackson.
MBI-Human Services Survey: Copyright ©1981 Christina Maslach & Susan E. Jackson.
MBI-Educators Survey: Copyright ©1986 Christina Maslach, Susan E. Jackson & Richard L. Schwab.
All rights reserved in all media. Published by Mind Garden, Inc., www.mindgarden.com

教學工作環境量表

填答者的資料

___性別　　1=男　　2=女

___年齡

1=25或25歲以下	4=36~40歲
2=26~30歲	5=41歲~
3=31~35歲	

___教育程度

1=高中（專科）肄業	5=研究所肄業
2=高中畢業	6=研究所碩士班畢業
3=大學或獨立學院肄業	7=研究所博士班畢業
4=大學或獨立學院畢業	

___任教年資

1=一年以下	4=11~15年
2=1~5年	5=16~25年
3=6~10年	6=26年及以上

目前職務是________________

For use by Wenshu Cao only. Received from Mind Garden, Inc. on April 24, 2024

這項問卷調查的目的是想了解教師們對其工作的看法。

在下列22個問題中，有一些有關工作情況的陳述，請仔細閱讀每一題句，看一看你是否對你的工作有那樣的感受。如果『從來沒有』那種感受，就請在該句前的短線上填“0”。若是你有過那種感受，就請用數字（由1~6）來表明經常性。

0=從來沒有
1=一年中有少數幾次或更少
2=每月一次或更少
3=每月有少數幾次
4=每周一次
5=每周有幾次
6=每日都有

___A1、我覺得我的工作使我心神耗損。
___A2、在每天工作完了時，我覺得精疲力竭。
___A3、我早上起來時就覺得疲倦，而又要面對一天的工作。
___A4、我很容易瞭解學生們對事情的想法。
___A5、我覺得我對待某些學生就像他們和我不相干一樣。
___A6、整天和人打交道對我眞是一件苦事。
___A7、我很有效地處理學生們的問題。
___A8、我對我的工作感到倦怠。
___A9、我覺得透過我的工作對別人的生活有正面的影響
___A10 從我擔任現在的工作後，我對人較爲冷漠。
___A11 我擔心這項工作在使我情緒麻木。
___A12 我覺得精力充沛。
___A13 我覺得我的工作使我有挫折感。
___A14 我覺得我做工作太賣力了。
___A15 我對某些學生的遭遇並不眞正關心。
___A16 和人們直接打交道對我的壓力很大。
___A17 我能很容易和學生之間建立輕鬆氣氛。
___A18 在和學生很接近地工作後，我覺得高興。
___A19 在目前工作上，我完成了一些有意義的事情。
___A20 我覺得我已經是無能爲力了。
___A21 在工作上，我很冷靜地應付情緒方面的問題。
___A22 我覺得學生們在某些問題上責怪我。

MBI-General Survey: Copyright ©1996 Wilmar B. Schaufeli, Michael P. Leiter, Christina Maslach & Susan E. Jackson.
MBI-Human Services Survey: Copyright ©1981 Christina Maslach & Susan E. Jackson.
MBI-Educators Survey: Copyright ©1986 Christina Maslach, Susan E. Jackson & Richard L. Schwab.
All rights reserved in all media. Published by Mind Garden, Inc., www.mindgarden.com

For use by Wenshu Cao only. Received from Mind Garden, Inc. on April 24, 2024

一、修訂教學工作感受問卷（MBI-Form ED）

作答說明：

(1)這份問卷的目的是在瞭解您實際教學工作以來，可能產生主觀的工作感受。

(2)每一題目代表一種主觀的工作感受，請您閱讀每一個題目，假如您沒有此種工作感受，請您在答案紙上" 0 "的位置打" √ "，假如您有此種感受，請您選一數目（1～6）最能代表您的感受。1：一年有幾次；2：每學期有幾次；3：每月有幾次；4：每兩週有幾次；5：每週有幾次；6：每天都有。

例題：我覺得我有職業倦怠症

0	1	2	3	4	5	6
			√			

說明：假如您對此題的感受選3，即表示您大概每個月都會有幾次職業倦怠的感受。

(3)請您依據實際工作的感受填答，不必在每個題花費時間去思考。

(4)全問卷共22題，請回答每一題目，不要有遺漏。

1.我覺得教學工作既吃力又乏味。
2.每到下班時間，我就感到疲憊不堪。
3.早上起床後，想到又須面對另一天的工作，我就會提不起精神。
4.我能輕易地瞭解學生對事物的感受（想法）。
5.我覺得我將某些學生視為不具人格的個體。
6.整天在學校與同事相處並教導學生，我覺得很辛苦。
7.我能有效地處理學生的問題。
8.我對教學工作感到厭倦。
9.我覺得我的教學工作能積極地影響學生。
10.從事教學工作以來，我對學生變得愈來愈冷淡。
11.我擔心教學工作會使我愈來愈冷漠無情。
12.我覺得精力充沛。
13.教學工作令我感到失望。
14.我覺得自己工作太認真。
15.我並不十分在意學生發生什麼事情。
16.教導學生和同事相處，會帶給我很大壓力。
17.我可以與學生融洽相處。
18.與學生一起工作，我就覺得很愉快。
19.在教學工作中，我完成許多有意義的事。
20.我對目前工作感到力不從心。
21.在教學工作中，我能很冷靜地處理情緒的問題。
22.我感到學生會為某些問題責怪我。

MBI-General Survey: Copyright ©1996 Wilmar B. Schaufeli, Michael P. Leiter, Christina Maslach & Susan E. Jackson.
MBI-Human Services Survey: Copyright ©1981 Christina Maslach & Susan E. Jackson.
MBI-Educators Survey: Copyright ©1986 Christina Maslach, Susan E. Jackson & Richard L. Schwab.
All rights reserved in all media. Published by Mind Garden, Inc., www.mindgarden.com

For use by Wenshu Cao only. Received from Mind Garden, Inc. on April 24, 2024

教师倦怠感问卷

多频繁

6	5	4	3	2	1	0
每一天	一星期几次	一星期一次	一个月几次	一个月一次或更少	一年几次或更少	从来没有

6	5	4	3	2	1	0	
___	___	___	___	___	___	___	1。我觉得我的工作热情已经耗尽。
___	___	___	___	___	___	___	2。一天工作下来,我感到筋疲力尽。
___	___	___	___	___	___	___	3. 每天早上起来,我仍感到疲劳,但还要面对新的一天的工作。
___	___	___	___	___	___	___	4。我能容易了解我的学生是如何感受他们周围的事物。
___	___	___	___	___	___	___	5。我感觉我没有把某些学生当作活生生的人来对待。
___	___	___	___	___	___	___	6。整天与学生打交道对我来说真是件头疼的事情。
___	___	___	___	___	___	___	7。我能非常有效地处理学生的问题。
___	___	___	___	___	___	___	8。我对工作有倦怠感。
___	___	___	___	___	___	___	9。我觉得我的工作能对他人产生积极的影响。
___	___	___	___	___	___	___	10.自从我从事这项工作以来,我变得对他人越来越冷漠。
___	___	___	___	___	___	___	11。我担心这个工作使我的情感变得麻木。
___	___	___	___	___	___	___	12。我觉得我的精力充沛。
___	___	___	___	___	___	___	13。我感到在工作中受到了挫折。
___	___	___	___	___	___	___	14。我觉得我对工作过于投入了。
___	___	___	___	___	___	___	15。我对某些学生的事情不大关心。
___	___	___	___	___	___	___	16。直接与其他人一起工作对我来说是一种很大的压力。
___	___	___	___	___	___	___	17。我能很容易与我的学生建立起一种轻松的气氛。
___	___	___	___	___	___	___	18。在与我的学生一起密切地工作之后,我感到很愉快。
___	___	___	___	___	___	___	19。在这项工作中,我完成了许多有价值的事情。
___	___	___	___	___	___	___	20。我觉得我智穷力竭。
___	___	___	___	___	___	___	21。在我的工作中,我能很平静地处理情感问题。
___	___	___	___	___	___	___	22。我觉得某些学生由于他们自己的一些问题而埋怨我.

谢谢您的作答.

MBI-General Survey: Copyright ©1996 Wilmar B. Schaufeli, Michael P. Leiter, Christina Maslach & Susan E. Jackson.
MBI-Human Services Survey: Copyright ©1981 Christina Maslach & Susan E. Jackson.
MBI-Educators Survey: Copyright ©1986 Christina Maslach, Susan E. Jackson & Richard L. Schwab.
All rights reserved in all media. Published by Mind Garden, Inc., www.mindgarden.com

For use by Wenshu Cao only. Received from Mind Garden, Inc. on April 24, 2024

The Chinese version of MBI-ES (2002)

指示

以下是關於輔導工作上的一些感受及輔導老師所持態度的描述。請小心細閱下列各題，根據以下指示，圈出一個最能形容你的數字。

0=從不發生 1=一年數次或更少 2=一月一次或更少 3=一月一次 4=一週一次 5=一週數次 6=每天皆發生

	從不發生	一年數次或更少	一月一次或更少	一月一次	一週一次	一週數次	每天皆發生
1. 我感到輔導工作使我熱情減退。	0	1	2	3	4	5	6
2. 一天的工作使我感到筋疲力盡。	0	1	2	3	4	5	6
3. 每早起來要面對新一天的工作，使我已感到疲乏。	0	1	2	3	4	5	6
4. 我很能明白學生對事物的感受。	0	1	2	3	4	5	6
5. 我感到我對待學生有如當他們是非人化的物體。	0	1	2	3	4	5	6
6. 要整天與人接觸共處對我實在是一項負擔。	0	1	2	3	4	5	6
7. 我很有效地處理學生問題。	0	1	2	3	4	5	6
8. 我對工作失去熱忱。	0	1	2	3	4	5	6
9. 我感到我能透過工作，正面地影響別人的一生。	0	1	2	3	4	5	6
10. 自擔任輔導工作後，我對人已變得冷漠麻木。	0	1	2	3	4	5	6
11. 我擔心輔導工作已使我的感情麻木。	0	1	2	3	4	5	6
12. 我覺得精力充沛。	0	1	2	3	4	5	6
13. 我的職業使我感到挫敗。	0	1	2	3	4	5	6
14. 我感到我太辛勞工作。	0	1	2	3	4	5	6
15. 我真的不在乎甚麼事情發生在一些學生身上。	0	1	2	3	4	5	6
16. 要與人共事實在給予我太大的壓力。	0	1	2	3	4	5	6
17. 我與學生共處可以很容易營造輕鬆的氣氛。	0	1	2	3	4	5	6
18. 我與學生緊密地相處後，我常感到很輕鬆愉快。	0	1	2	3	4	5	6
19. 我在工作上有很多有意義的成就。	0	1	2	3	4	5	6
20. 我感到好像有身臨絕境的感覺。	0	1	2	3	4	5	6
21. 在工作上我能冷靜地處理問題。	0	1	2	3	4	5	6
22. 我覺得學生將他們的問題歸咎於我。	0	1	2	3	4	5	6

MBI-General Survey: Copyright ©1996 Wilmar B. Schaufeli, Michael P. Leiter, Christina Maslach & Susan E. Jackson.
MBI-Human Services Survey: Copyright ©1981 Christina Maslach & Susan E. Jackson.
MBI-Educators Survey: Copyright ©1986 Christina Maslach, Susan E. Jackson & Richard L. Schwab.
All rights reserved in all media. Published by Mind Garden, Inc., www.mindgarden.com

For use by Wenshu Cao only. Received from Mind Garden, Inc. on April 24, 2024

MBI-HSS Traditional Chinese Version

1.我的工作把我的精神榨乾了
2.工作一天下來讓我感到精疲力盡
3.一大早起來，想到又要面對一天的工作，使我感到很疲倦
4.我可以很容易瞭解病人的感受
5.我會把某些病人當成沒有感覺的東西
6.在工作中整天和人來往，使我感到精神緊繃
7.我可以很有效地解決病人的問題
8.我對我的工作感到倦怠
9.我覺得我的工作可以使其他人過得更好
10.從事這個工作以來，我對待病人越來越不帶感情
11.我擔心這個工作會使我變得越來越冷酷
12.我覺得精力充沛
13.我的工作給我很大的挫折
14.我覺得我工作太賣力了
15.我並不是真的關心病人究竟發生了什麼事
16.直接面對病人的工作帶給我很大的壓力
17.跟病人在一起的時候，我很容易營造一個輕鬆的氣氛
18.每當和病人密切合作之後，我感到精神愉悅
19.我在這個工作當中，完成了許多有意義的事
20.在工作上，我感到身心俱疲
21.在我的工作當中，我可以很冷靜地處理情緒問題
22.我覺得病人會將他們所遭遇的一些問題怪罪於我

TA-218 – MBI-HSS – Traditional Chinese – All 22 items

MBI-General Survey: Copyright ©1996 Wilmar B. Schaufeli, Michael P. Leiter, Christina Maslach & Susan E. Jackson.
MBI-Human Services Survey: Copyright ©1981 Christina Maslach & Susan E. Jackson.
MBI-Educators Survey: Copyright ©1986 Christina Maslach, Susan E. Jackson & Richard L. Schwab.
All rights reserved in all media. Published by Mind Garden, Inc., www.mindgarden.com

For use by Wenshu Cao only. Received from Mind Garden, Inc. on April 24, 2024

与工作的关系

下面总共有16项描述，请您根据自己的感受和体会，判断它们在您所在的公司或者您身上发生的频率。如果您从来没有这种想法或体会，请选择0；如果您曾经有这种想法或体会，请选择合适的数字。

	1	2	3	4	5	6
从	极少<br>一年几次或更	偶尔<br>一个月一次或者更	经常<br>一个月几	频繁<br>每星期一	非常频<br>一星期几	每天

1.	工作让我感觉身心俱惫							
2.	下班的时候我感觉精疲力竭							
3.	早晨起床时，我感觉非常累，可是又不得不去面对一天的工作。							
4.	整天工作对我来说确实压力很大							
5.	我能有效地解决工作中出现的问题							
6.	工作让我有快要崩溃的感觉							
7.	我觉得我做的工作有益于公司的发展							
8.	自从开始干这份工作，我对工作越来越不感兴趣							
9.	我对工作不象以前那样热心了							
10.	在我看来，我擅长于自己的工作							
11.	当完成工作任务时，我感到非常高兴							
12.	我完成了很多有价值的工作							
13.	我只希望不受影响地干自己的工作							
14.	我怀疑自己所做的工作的价值							
15.	我对自己所做的工作是否有贡献越来越不关心							
16.	我自信自己能有效地完成各项工作							

MBI-General Survey: Copyright ©1996 Wilmar B. Schaufeli, Michael P. Leiter, Christina Maslach & Susan E. Jackson.
MBI-Human Services Survey: Copyright ©1981 Christina Maslach & Susan E. Jackson.
MBI-Educators Survey: Copyright ©1986 Christina Maslach, Susan E. Jackson & Richard L. Schwab.
All rights reserved in all media. Published by Mind Garden, Inc., www.mindgarden.com

For use by Wenshu Cao only. Received from Mind Garden, Inc. on April 24, 2024

**說明: 下列之 16 項敘述為您對在校情況的感受。請詳閱各項，並針對您是否曾在學業方面感覺如此加以確認。若未曾感覺如此，請於該項後填寫數字 0 (零)。若曾出現該種感受，請填寫數字(1 至 6)來確實表示感覺出現的頻率。**		未曾	一年內有幾次但不多	一個月一次或以下	一個月數次	每週一次	每週數次	每天如此
1.	我覺得被課業榨乾情緒。	0	1	2	3	4	5	6
2.	在學校一整天，結束時我感到精疲力盡。	0	1	2	3	4	5	6
3.	我感到疲憊，當早上起來就必須面對另一整天的學校生活。	0	1	2	3	4	5	6
4.	去課堂一整天對我來說實在是一種沉重的壓力。	0	1	2	3	4	5	6
5.	我可以有效地解決學習上出現的問題。	0	1	2	3	4	5	6
6.	我感到我的課業讓我疲憊不堪。	0	1	2	3	4	5	6
7.	我感覺我在課堂上做出了實質的貢獻。	0	1	2	3	4	5	6
8.	自從開學以來，我對學業的興趣減少了。	0	1	2	3	4	5	6
9.	我已變得對學業的熱情減少了。	0	1	2	3	4	5	6
10.	在我的想法裡，我是一個好學生。	0	1	2	3	4	5	6
11.	我感到十分振奮，當我在大學裡成就某件事時。	0	1	2	3	4	5	6
12.	我在學習中完成了許多有價值的事情。	0	1	2	3	4	5	6
13.	我只是想把我的學業完成，不要被困擾。	0	1	2	3	4	5	6
14.	我已變得對我的大學課業是否貢獻任何事感到更加憤世嫉俗。	0	1	2	3	4	5	6
15.	我懷疑我學習的意義。	0	1	2	3	4	5	6
16.	在大學學習時，我有信心能有效完成各項事務。	0	1	2	3	4	5	6

TA-869 – MBI-GS (S) – Taiwan Mandarin

MBI - General Survey for Students – MBI-GS (S): Copyright ©1996, 2016 Wilmar B. Schaufeli, Michael P. Leiter, Christina Maslach & Susan E. Jackson. All rights reserved in all media. Published by Mind Garden, Inc. www.mindgarden.com.
