# Supplementary material for: Changes in sleep patterns in primary care workers during the first wave of the COVID-19 pandemic in 2022 in Shanghai: a cross-sectional study
Source: Sci Rep. 2024 May 29;14:12373. doi: 10.1038/s41598-024-61311-z (PMC11137069; doi:10.1038/s41598-024-61311-z)
Supplement: Supplementary file 3 — Supplementary Information 3. [file 41598_2024_61311_MOESM3_ESM.pdf]

Sleep during the COVID-19 outbreak

Disclaimer: This is an anonymous questionnaire and participating primary health care providers can answer it with confidence!

As general practitioners in a community hospital in Shanghai, we noticed that the current pandemic in Shanghai h as had a major impact on the work and rhythms of primary care health care providers such as community general practitioners, nurses, administration and logistics. We conducted an inquiry to investigate the sleep and perception s of primary care health care providers in different community hospitals in Shanghai during the pandemic, and in t his way possibly find suitable improvements in the work and routine of primary care providers under a long-term p andemic policy, now or in the future.We achieved our goal by means of an online questionnaire. This survey is sho rt, voluntary and anonymous. Your information will not be associated with your name and the results of the survey will not affect your life. Likewise, we do not follow up the results of the study after they have been generated.

1.Please describe your role/job in the community hospital where you work *

○ Physician

○ Clinical Nurse

○ Pharmacist

○ Logisticians

○ Administrative staff

○ Other (not listed in the table above) ________________

2.Where have you worked during this pandemic [多选题] *

If you have worked in more than one location, you can select more than one location.

□ Ward

□ Outpatient

□ Sealed and controlled cells

□ Isolation Hotel

□ Fangcang Hospital

□ Not listed above ________________

3.What is your current level *

◯ Junior

◯ Intermediate

◯ Senior

4.How often have you been exposed to new crown nucleic acid samples/high risk environment/confirmed COVID-19 positive cases *

◯ Never been exposed to

◯ At least 1 time, but less than 5 times

◯ Weekly contact

◯ Daily exposure

5.Have you (or a medical colleague around you) ever been diagnosed positive for COVID-19? *

This item is absolutely anonymous and you can choose with confidence!

◯ Yes

◯ No

6.Please describe how your work has changed in the current Shanghai COVID-19 outbreak [多选题] *

☐ No change

☐ Increased my working hours

☐ Reduced my working hours

☐ Changed where I work (e.g., from the ward to the community for long-term nucleic acid sampling, etc.)

☐ Changed the nature of my job (e.g., from administrative to medical duties or from logistical duties to nucleic acid testing duties, etc.)

☐ I mainly work from home now

☐ I have left my job

☐ Other ________________________________

7.Please select what difficulties this Shanghai outbreak has caused you. [多选题] *

- ☐ Did not bring difficulties
- ☐ Increased family responsibilities and load
- ☐ Occasional screenings or emergency assignments during an outbreak
- ☐ Worry about the difficulty of accessing medical care for you and your family in case of sudden illness
- ☐ Financial difficulties
- ☐ Emotional Stress
- ☐ Other ________________

8.How many children are in your family *

- ○ 0 pcs
- ○ 1pc
- ○ 2
- ○ 3
- ○ 4 and above

9.If you have children, please select your child's age category (skip this question if you do not have children) [多选题]

If you have more than two children, you can make multiple selections.

- ☐ Newborn or infant (<1 year)
- ☐ Preschoolers or toddlers (1-4 years old)
- ☐ School-age children (5-12 years old)
- ☐ Teenagers (12-18 years old)
- ☐ Adults (>18 years old)

10.Please answer the following questions

If you don't have children, please skip this question!

	Yes	No
Has this outbreak in Shanghai changed your daily management plan for your child	○	○
Whether there are other adults in the home (e.g., babysitters, grandparents) to help care for the child	○	○
Do you spend more than 15 minutes a day helping your child with school or educational tasks	○	○
Did you change your place of residence during the outbreak in Shanghai (e.g., concern about the risk of infection to children or the elderly)	○	○

11.Please indicate how often these problems below occurred during the current Shanghai outbreak (COVID-19). *

Please tick the last box if there is no change from before the outbreak COVID-19.

	Never	Almost never	Sometimes	Frequently often	very often	No change from before the outbreak
Distracted by something unpredictable happening	○	○	○	○	○	○
You start to feel like you have no control over the important things in your life	○	○	○	○	○	○
You will feel nervous and stressed	○	○	○	○	○	○
Begin to be unable to successfully deal with annoying life troubles	○	○	○	○	○	○
Feelings of beginning to be unable to deal effectively with the important changes that occur in your life	○	○	○	○	○	○
Feel a loss of confidence in dealing with your own personal problems	○	○	○	○	○	○
Feeling that things are not going well	○	○	○	○	○	○
Finding yourself unable to handle all the things you have to do	○	○	○	○	○	○
Inability to control the annoying things in life	○	○	○	○	○	○
Inability to be the master of things	○	○	○	○	○	○
often angry, because many things happen beyond their control	○	○	○	○	○	○
Often avoiding the things you think you must	○	○	○	○	○	○

accomplish						
Unable to grasp how to organize time	○	○	○	○	○	○
Often feel that difficult things are piling up and you can't overcome them	○	○	○	○	○	○
You have no interest or pleasure in doing things	○	○	○	○	○	○
You feel depressed, overwhelmed and helpless	○	○	○	○	○	○

12.请回答以下问题 *

	Never	Occasionally	1-2 times a month	2-3 times a month	1 time a week	2-4 times a week	Daily
How many times has the thought crossed your mind that since the pandemic began, I have become more callous to others?	○	○	○	○	○	○	○
How many times have you had the thought in your head that I feel exhausted from my work	○	○	○	○	○	○	○

13.Prior to the Shanghai COVID-19 outbreak, were you diagnosed, treated, or suspected of having any of the following conditions? [多选题] *

Multiple options available

☐ Anxiety

☐ Depression

☐ Insomnia

☐ Sleep apnea or snoring, pauses in breathing

☐ No above symptoms

14.Before the Shanghai epidemic, please describe each aspect of your sleep listed below. *

	Never	Very little (1-2 times a month)	Sometimes	Frequently (2-3 times per week)	Very frequent (4-5 times per week)	All the time (basically every day)
You have a fixed frequency of sleep rhythms (i.e. going to bed at the same time of night and waking up at the same time in the morning)	○	○	○	○	○	○
The frequency with which you wake up refreshed	○	○	○	○	○	○
You can stay awake all day without nodding off or dozing off as often	○	○	○	○	○	○
How often you go to bed (or try to fall asleep) before 12:00 midnight	○	○	○	○	○	○
The frequency of the interval between the onset of sleep and your falling asleep <30 minutes (including a brief awakening during the night and then falling asleep again)	○	○	○	○	○	○
You have 6-8 hours of sleep per day	○	○	○	○	○	○
How often you have nightmares (or dreams that cause anxiety, fear and bad feelings)	○	○	○	○	○	○

15.During the Shanghai outbreak, please describe each aspect of your sleep listed below. *

Please note the distinction from the 👆 above question！！！！

	Never	Very little (1-2 times a month)	Sometimes	Frequently (2-3 times per week)	Very frequent (4-5 times per week)	All the time (basically every day)
You have a fixed frequency of sleep rhythms (i.e. going to bed at the same time of night and waking up at the same time in the morning)	○	○	○	○	○	○
The frequency with which you wake up refreshed	○	○	○	○	○	○
You can stay awake all day without nodding off or dozing off as often	○	○	○	○	○	○
How often you go to bed (or try to fall asleep) before 12:00 midnight	○	○	○	○	○	○
The frequency of the interval between the onset of sleep and your falling asleep < 30 minutes (including a brief awakening during the night and then falling asleep again)	○	○	○	○	○	○
You have 6-8 hours of sleep per day	○	○	○	○	○	○
How often you have nightmares (or dreams that cause anxiety, fear and bad feelings)	○	○	○	○	○	○

16.What is the biggest change in overall sleep patterns that you have noticed since the start of the Shanghai outbreak (COVID-19)? *

单选题

○ My sleep has not changed

○ It is difficult to fall asleep or stay asleep well

○ I doze or nod off more frequently during the day

○ My sleep schedule has changed (e.g., I'm sleeping later)

○ My total amount of sleep has increased or decreased

○ Other ____________________

17.During the Shanghai outbreak, you may change your daytime habits, which can have an impact on your sleep. Here are a few examples of these. *

If this is the same as before the pandemic, tick the last box.

	Never	Occasionally, only 1-2 times	Sometimes, 1-2 times a week	Frequently, 3-4 times a week	Very frequent, basically every day	Always, every day	No change from before the outbreak
Caffeine (including Red Bull, iced tea, cola, coffee) within 12 hours before bedtime	○	○	○	○	○	○	○
Drinking alcohol within 6 hours before bedtime	○	○	○	○	○	○	○
Smoking within 4-6 hours before bedtime	○	○	○	○	○	○	○
Watching/using cell phones, tablets or laptops at night	○	○	○	○	○	○	○

18.Here are the final questions. Knowing your basic information will help us put your answers into context. Your age: ________

These questions are optional, not mandatory. We will respect and protect all your privacy!

19.Your gender

◯ Male

◯ Female

20.Your place of origin

◯ Shanghai nationality

◯ Non-Shanghai

21.Describe your personal status

◯ Single

◯ Unmarried but have boyfriend/girlfriend

◯ Married

◯ Divorce status

Copyright © 1998-2023 Tencent.
系统由腾讯问卷提供用户协议服务协议隐私政策
