# Supplementary material for: Changes in sleep patterns in primary care workers during the first wave of the COVID-19 pandemic in 2022 in Shanghai: a cross-sectional study
Source: Sci Rep. 2024 May 29;14:12373. doi: 10.1038/s41598-024-61311-z (PMC11137069; doi:10.1038/s41598-024-61311-z)
Supplement: Supplementary file 4 — Supplementary Information 4. [file 41598_2024_61311_MOESM4_ESM.pdf]

2022年上海新冠疫情期间社区医护睡眠调查

声明：此问卷为匿名问卷，参与调查的初级卫生保健医疗服务人员可放心作答！

作为上海某社区医院的全科医生，我们注意到本次上海疫情大流行对社区全科医生、护士、行政、后勤等初级保健的医疗服务人员的工作和生活节律产生了较大影响。我们进行了一项探究，以调查大流行期间上海不同社区医院初级保健医疗服务人员的睡眠情况和感觉，通过这种方式可能在现在或将来，为长期疫情政策下初级保健人员的作息寻找合适的改善方法。

我们通过在线问卷调查的形式来实现我们的目标。这项调查是简短的，自愿的和匿名的。您的信息不会和您的姓名相关联，调查结果也不会影响到您的生活。同样，在研究结果产生后我们也不会作后续跟踪调查。

1.请描述您在工作的社区医院中担任的角色/工作 *

○ 临床医生

○ 临床护士

○ 药剂科医生

○ 后勤人员

○ 行政人员

○ 其他（未在上表中列出）________________

2.在本次大流行期间，您在哪些地方工作过 [多选题] *

如果您在多个地方工作过，可多选多个地点。

☐ 病房

☐ 门诊

☐ 封控小区

☐ 隔离酒店

☐ 方仓医院

☐ 以上未列出________________________

3.您目前的职称是什么 *

○ 初级

○ 中级

○ 高级

4.您多长时间接触过新冠核酸样本/高风险环境/确诊的新冠（COVID-19）阳性病例 *

○ 从没接触过

○ 至少1次，但是小于5次

○ 每周都有接触

○ 每天都接触

5.您（或您周围的医疗同事）有确诊过新冠（COVID-19）阳性吗？ *

此项是绝对匿名的，您可以放心选择！

○ 有

○ 没有

6.请描述你的工作在本次上海新冠COVID-19疫情中有什么变化 [多选题] *

☐ 没有变化

☐ 增加了我的工作时间

☐ 减少了我的工作时间

☐ 改变了我的工作地点（如从病房到社区进行长期核酸采样等）

☐ 改变了我的工作性质（如从行政职责转到医疗职责或从后勤职责转到核酸检测职责等）

☐ 我现在主要在家工作

☐ 我已经离职了

☐ 其他________________

7.请选择本次上海疫情给您带来了哪些困难？ [多选题] *

☐ 没有带来困难

☐ 家庭责任增加和负荷加重

☐ 疫情期间不定时的筛查或紧急任务

☐ 担心您和您的家人突发疾病就医困难

☐ 经济困难

☐ 情感压力

☐ 其他________________

8.您的家庭里有几个孩子 *

○ 0个

○ 1个

○ 2个

○ 3个

○ 4个及以上

9.如果您有孩子，请选择您孩子的年龄类别**（没有孩子请跳过此题）** [多选题]

如果您有两个以上的孩子，可进行多选。

☐ 新生儿或婴儿（＜1岁）

☐ 学龄前儿童或幼儿（1-4岁）

☐ 学龄儿童（5-12岁）

☐ 青少年（12-18岁）

☐ 成人（＞18岁）

10.请您回答一下问题

如果您没有孩子，请跳过此题！

	是	否
本次上海疫情是否改变了您对孩子的日常管理计划	○	○
家中是否有其他成年人（如保姆，爷爷奶奶）帮助照顾孩子	○	○
您是否每天花15分钟以上的时间来帮助你的孩子完成学业或教育任务	○	○
在上海疫情期间，您是否改变了您的居住地点（如担心孩子或老人被传染风险）	○	○

11.请说明以下这些问题在本次上海疫情（COVID-19）期间发生的频率。 *

如果与疫情COVID-19**之前**相比没有变化，请**勾选**最后一个框。

	从来没有	几乎没有	有时会	比较频繁	特别频繁	**与疫情发生之前没有变化**
一些无法预期的事情发生而感到心烦意乱	○	○	○	○	○	○
你开始觉得你无法控制你生活中的重要的事情	○	○	○	○	○	○
你会感到紧张和压力	○	○	○	○	○	○
开始无法成功地处理恼人的生活麻烦	○	○	○	○	○	○
感到开始无法有效地处理生活中所发生的重要改变	○	○	○	○	○	○
对于处理自己私人的问题感到丧失信心	○	○	○	○	○	○
感到诸事不顺	○	○	○	○	○	○
发现自己无法处理所有自己必须做的事情	○	○	○	○	○	○
无法控制生活中恼人的事情	○	○	○	○	○	○
无法成为驾驭事情的主人	○	○	○	○	○	○
常生气，因为很多事情的发生是超出自己所能控制的	○	○	○	○	○	○
经常逃避想到的自己必须完成的事情	○	○	○	○	○	○
无法掌握时间的安排方式	○	○	○	○	○	○
常感到困難的事情堆积如山，而自己无法克服他们	○	○	○	○	○	○
你对做事情没有兴趣和乐						

趣	○	○	○	○	○	○
你感到沮丧、压抑和无助	○	○	○	○	○	○

12.请回答以下问题 *

	从来没有	偶尔	一个月1-2次	一个月2-3次	一周1次	一周2-4次	每天
您有多少次脑海里产生这样的想法：自从大流行病开始以来，我对别人变得更加冷酷无情了。	○	○	○	○	○	○	○
您有多少次脑海里产生这样的想法：我觉得自己的工作很疲惫	○	○	○	○	○	○	○

13.在上海新冠（COVID-19）疫情之前，你是否曾被诊断、治疗或怀疑自己有以下情况？ [多选题] *

可多选

☐ 焦虑

☐ 抑郁

☐ 失眠

☐ 睡眠呼吸暂停或打鼾，呼吸停顿

☐ 没有以上症状

14.上海疫情**之前**，请描述下面列出的你的睡眠的每个方面。 *

	从来没有	极少（一个月1-2次）	有时	经常（每周2-3次）	很频繁（每周4-5次）	一直是这样（基本每天）
你有一个固定的睡眠时间节律的频率（即在晚上同一时段上床和早上同一时段醒来）	○	○	○	○	○	○
你在醒来时神清气爽的频率	○	○	○	○	○	○
您可以整天保持清醒且不打盹和打瞌睡的频率	○	○	○	○	○	○
您在半夜12点前睡觉（或试图睡着）的频率	○	○	○	○	○	○
您从开始酝酿睡意到进入睡眠状态间隔＜30分钟的频率（包括夜起短暂清醒后再次进入睡眠状态）	○	○	○	○	○	○
您每天有6-8小时睡眠的频率	○	○	○	○	○	○
您做噩梦（或引起焦虑，恐惧和不良情绪的梦）的频率	○	○	○	○	○	○

15.上海疫情**期间**，请描述下面列出的你的睡眠的每个方面。 *

请注意与👆上题区分！！！！

	从来没有	极少（一个月1-2次）	有时	经常（每周2-3次）	很频繁（每周4-5次）	一直是这样（基本每天）
你有一个固定的睡眠时间节律的频率（即在晚上同一时段上床和早上同一时段醒来）	○	○	○	○	○	○
你在醒来时神清气爽的频率	○	○	○	○	○	○
您可以整天保持清醒且不打盹和打瞌睡的频率	○	○	○	○	○	○
您在半夜12点前睡觉（或试图睡着）的频率	○	○	○	○	○	○
您从开始酝酿睡意到进入睡眠状态间隔＜30分钟的频率（包括夜起短暂清醒后再次进入睡眠状态）	○	○	○	○	○	○
您每天有6-8小时睡眠的频率	○	○	○	○	○	○
您做噩梦（或引起焦虑，恐惧和不良情绪的梦）的频率	○	○	○	○	○	○

16.自上海疫情（COVID-19）开始以来，你注意到的整体睡眠模式的**最大变化**是什么？ *

单选题

○ 我的睡眠没有发生变化

○ 入睡或保持良好睡眠状态很困难

○ 我更频繁地在白天打盹或打瞌睡

○ 我的睡眠时间发生了变化（如：我睡得更晚了）

○ 我的睡眠总量增加或减少了

○ 其他________________________________

17.在上海疫情期间，你可能会改变白天的习惯，这对你的睡眠有影响。以下是其中几个例子。 *

如果这与大流行之前的情况相同，请勾选最后一个框。

	从来没有	偶尔，只有1-2次	有时，每周1-2次	经常，一周3-4次	很频繁，基本每天	一直，每天都这样	**跟疫情之前没有变化**
睡觉前12小时内喝咖啡因（包括红牛、冰茶、可乐、咖啡）	○	○	○	○	○	○	○
睡前6小时内饮酒	○	○	○	○	○	○	○
睡前4-6小时内抽烟	○	○	○	○	○	○	○
晚上看/使用手机、平板电脑或笔记本电脑	○	○	○	○	○	○	○

18.以下是最后的问题啦。知道您的基本信息将有助于我们把您的答案纳入的背景。
您的年龄：__________岁

这些问题是可选择的，而不是必答的。我们将尊重和保护您的一切隐私！

19.您的性别

○ 男

○ 女

20.您的籍贯

○ 上海籍

○ 非上海籍

21.描述您的个人状态

○ 单身

○ 未婚但有男（女）朋友

○ 已婚

○ 离异状态

Copyright © 1998-2022 Tencent.
系统由腾讯问卷提供用户协议服务协议隐私政策
